# Active graphene–silicon hybrid diode for terahertz waves

**DOI:** 10.1038/ncomms8082

**Published:** 2015-05-11

**Authors:** Quan Li, Zhen Tian, Xueqian Zhang, Ranjan Singh, Liangliang Du, Jianqiang Gu, Jiaguang Han, Weili Zhang

**Affiliations:** 1 Center for Terahertz Waves and College of Precision Instrument and Optoelectronics Engineering, Tianjin University and the Key Laboratory of Optoelectronics Information and Technology (Ministry of Education), Tianjin 300072, China; 2 School of Electrical and Computer Engineering, Oklahoma State University, Stillwater, Oklahoma 74078, USA; 3 Division of Physics and Applied Physics, Center for Disruptive Photonic Technologies, School of Physical and Mathematical Sciences, Nanyang Technological University, 21 Nanyang Link, Singapore 637371, Singapore

## Abstract

Controlling the propagation properties of the terahertz waves in graphene holds great promise in enabling novel technologies for the convergence of electronics and photonics. A diode is a fundamental electronic device that allows the passage of current in just one direction based on the polarity of the applied voltage. With simultaneous optical and electrical excitations, we experimentally demonstrate an active diode for the terahertz waves consisting of a graphene–silicon hybrid film. The diode transmits terahertz waves when biased with a positive voltage while attenuates the wave under a low negative voltage, which can be seen as an analogue of an electronic semiconductor diode. Here, we obtain a large transmission modulation of 83% in the graphene–silicon hybrid film, which exhibits tremendous potential for applications in designing broadband terahertz modulators and switchable terahertz plasmonic and metamaterial devices.

## Abstract (web summary)

Graphene has demonstrated the ability to modulate terahertz (THz) waves by optical or electrical excitation, but modulation depths have been low. Here, Li *et al*. demonstrate enhanced modulation and polarity-dependent THz attenuation using external voltage bias and photoexcitation on a graphene–silicon film.

## Graphical abstract

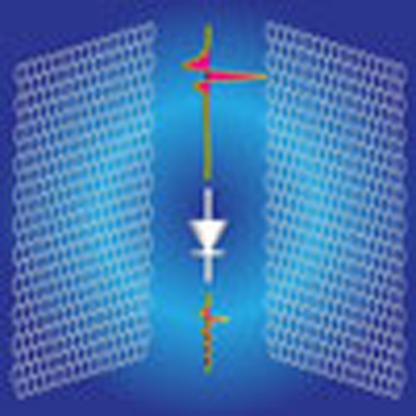

Over the past few years, graphene, as a two-dimensional version of graphite, has attracted a lot of attention from physicists, engineers and material scientists. Graphene also holds great promise for photonic and optoelectronic device applications^1^,^2^,^3^. Owing to the special arrangement of atoms in graphene, that is, the hexagonal lattice, a Dirac cone-type electronic band structure is formed over a wide range of energies so that the electrons behave as massless Dirac fermions. Many exotic physical phenomena have been observed in graphene, such as anomalous quantum Hall effect, extremely high carrier mobility, saturable absorption, optical amplification and tunable interband and intraband conductivities^4^,^5^,^6^,^7^,^8^,^9^. Such intriguing properties make graphene a very promising material in many potential applications ranging from new type of electronic elements and quantum computers to energy storage devices and optical modulators.

In the terahertz regime, graphene is also one of the most prosperous materials in modulating the propagation properties of the terahertz wave. One way to realize such modulation is to apply external voltage on the graphene–insulator–semiconductor structures, so that the Fermi level in graphene could be tuned accordingly and thus the optical conductivity due to the intraband transition process or free carrier absorption^10^,^11^,^12^,^13^,^14^,^15^. However, in most previous studies, the modulation depth was quite low due to limited carrier injection even at higher values of externally applied voltage bias^11^,^12^,^13^. Recently, it has been reported that by integrating graphene with metamaterials, significant transmission modulation of up to ∼47% was achieved with grating gated graphene metamaterials^14^. Another route to achieve higher modulation of terahertz signals using graphene-based hybrid systems could be to optically pump the graphene–semiconductor hybrid structures, where the photogenerated carriers in the semiconductor substrate would directly diffuse into graphene, thus altering the terahertz properties and enabling a significant modulation depth^16^,^17^,^18^. However, graphene-based terahertz modulators have seldom been studied under simultaneous influence of both the external voltage bias and optical pump excitation.

In this study, we investigate the impact of dual excitation behaviour of a graphene–silicon hybrid film where we achieve enhanced modulation of the terahertz waves at low voltage bias, that is, a few volts and low fluence photoexcitation. We discover that the transmission modulation of the terahertz waves could be achieved under different polarities of the voltage bias and simultaneous photoexcitation with a continuous wave (CW) green laser (532 nm). Moreover, on photoexcitation, the graphene–silicon hybrid film transmits the terahertz waves when biased with a positive voltage, whereas it blocks the wave when biased with a negative voltage, resembling the characteristic of a typical semiconductor-based electronic diode that allows passage of current only for positive bias and blocks the current when negatively biased. Thus, the proposed graphene–silicon hybrid film could function as ‘a diode' for the terahertz waves, which we would address from here on as graphene–silicon terahertz diode (GSTD).

## Results

### Sample preparation

**Figure 1:**
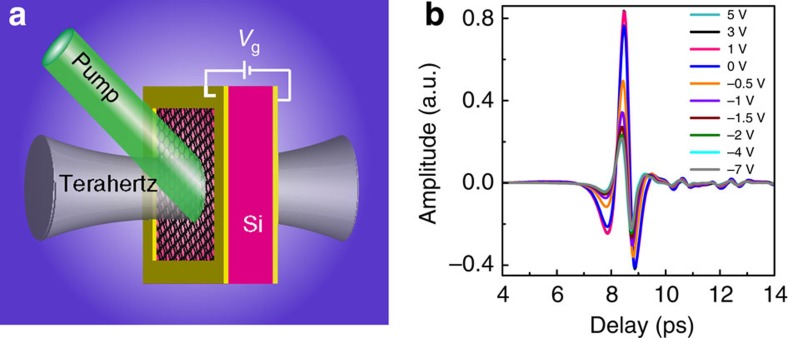
Experimental design and normalized time-domain signals. (**a**) Illustration of the GSTD sample. The double-layer graphene on the silicon substrate was photoexcited with green light and biased with voltage 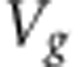. (**b**) Normalized terahertz time-domain signals at various gate bias voltages under a photoexcitation power of 280 mW.

The proposed GSTD is schematically illustrated in Fig. 1a. A large-area graphene monolayer was first grown on copper by chemical vapour deposition, using methane and hydrogen^19^. A thin layer of poly-(methyl methacrylate) was then coated onto the monolayer. In the next step, two layers of graphene were transferred onto a 510-μm-thick high-resistivity silicon substrate (*N*-type, resistivity *ρ*>8,000 Ω cm) in sequence, by using wet etching method to remove the copper and poly-(methyl methacrylate), respectively^20^,^21^. The randomly stacked monolayer graphene still behaved as an isolated monolayer graphene owing to electrical decoupling between the layers^22^,^23^. The quality of the graphene samples was characterized and verified by Raman spectroscopy^24^,^25^ (Supplementary Fig. 1). The entire sample area is 1 cm^2^. The double-layer graphene was chosen due to enabled higher modulation depth and improved efficiency in terms of diode performance when compared with the monolayer counterpart (Supplementary Fig. 2). As shown in Fig. 1a, two square metallic rings were carefully placed onto both the graphene and silicon sides as two electrodes, to bias the graphene on silicon. Meanwhile, a CW green laser was used to illuminate the sample.

### Sample characterization

**Figure 2:**
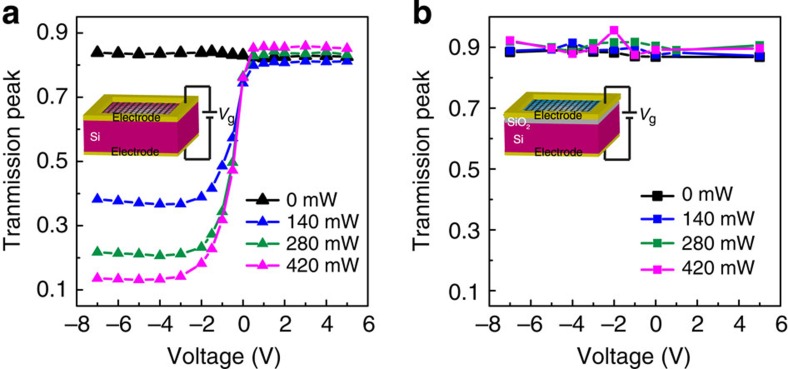
Gate- and photoexcitation-controlled amplitude change of normalized time-domain transmission peaks. Gate voltage-dependent, normalized time-domain transmission peaks of the double-layer graphene (**a**) on silicon and (**b**) on SiO_2_-silicon substrate at different laser photoexcitation power, respectively. Insets: schematics of the measured devices.

A terahertz time-domain spectroscopy^26^,^27^ system was used to measure the transmission spectra of the GSTD sample under different optical pump illumination power *P* and bias voltages 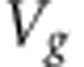 at normal incidence. As a reference, an identical bare piece of silicon without the graphene layer was photoexcited with the same optical power without bias voltage. Figure 1b shows the measured normalized time-domain signal of the GSTD sample biased with different voltages under a CW optical illumination power *P*=280 mW. It was observed that the bias voltage caused significant changes in the peak amplitude of the terahertz pulse. A similar behaviour was observed at varied pump power. Figure 2a represents the normalized peak values of the time-domain signals from the GSTD sample biased from −7 to 5 V under different CW optical illumination power *P*=0, 140, 280 and 420 mW, respectively. Without photoexcitation, the applied bias voltage did not cause any significant change in the terahertz transmission through the GSTD sample. However, when the biased graphene–silicon hybrid film was photoexcited, we observed transmission modulation. At positive bias, the increase in voltage (under fixed photoexcitation) led to a negligible change in the transmission, whereas the increase in the photoexcitation power (with fixed positive bias) caused a visible change, corresponding to a modulation depth of <12%. The modulation depth is defined as: 
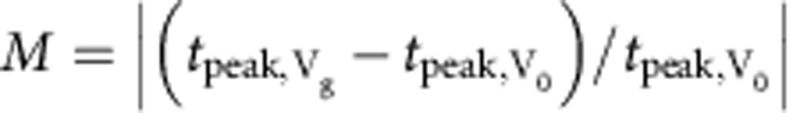
, where 
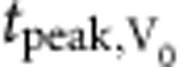
 and 
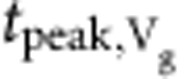
 are the normalized time-domain transmission peaks for zero and 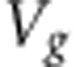 gate voltages, respectively. On the contrary, at negative bias, drastic decrease in the terahertz transmission was observed with increase in negative bias voltage. As shown in Fig. 2a, under the illumination power *P*=140 mW, modulation depth of up to 51% was achieved when the bias was changed from 0 to −3 V. With further increase in the illumination power *P* to 420 mW, the modulation depth was increased to a maximum value of 83% when −4 V bias was applied. The terahertz transmission reached a saturation point at a negative bias voltage of approximately −3 V for the three different levels of photoexcitation. Besides, the dynamic electrical behaviour of the GSTD device was characterized. By applying an AC rectangular voltage at various frequencies, the modulation speed was observed to be around 1 kHz (Supplementary Fig. 3).

In such a graphene–silicon hybrid film, we attribute the observed ‘diode' characteristics to the variation in carrier density in the graphene layer due to the hybrid graphene–silicon interface. As a control experiment, we also prepared another sample in which the double-layer graphene was transferred onto a silicon substrate with a 300-nm-thick top SiO_2_ layer. Thus, the graphene film was separated from the silicon substrate by the SiO_2_ spacer. When the same experiment condition was repeated with this control sample, we did not observe any modulation effect, as shown in Fig. 2b. Previous work with the SiO_2_ spacer layer reported that tens of volts of applied bias would cause small modulations (lower than 20%) on the transmitted terahertz waves^12^,^13^,^15^. In our experiment, however, the low-voltage bias did not result in any obvious modulation of the transmission signal.

### Theoretical calculations

**Figure 3:**
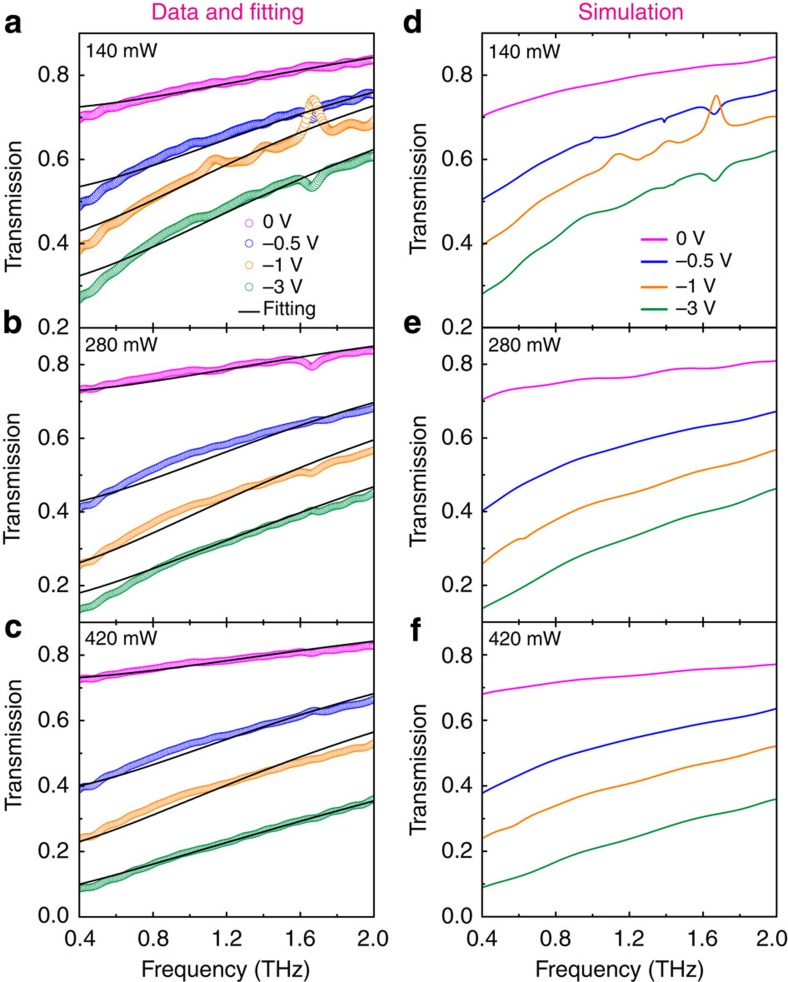
Gate- and photoexcitation-controlled amplitude change of frequency-domain transmission. (**a**–**c**) Measured (circle) and theoretical (solid black curve) values of the transmission amplitude as a function of frequency at gate biases of 0, −0.5, −1 and −3 V under photoexcitation power at *P*=140, 280 and 420 mW, respectively. (**d**–**f**) Plots of simulation results with thin film approximation corresponding to **a**–**c**, respectively.

To acquire further understanding of the ‘diode' characteristics, we now discuss the carrier properties of the graphene layer under optical illumination and electrical biasing. Based on the terahertz time-domain spectroscopy measurements, the frequency-dependent amplitude transmission was obtained by 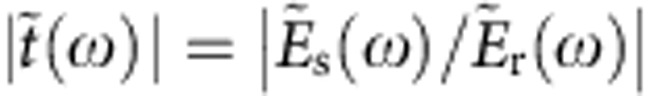, where 
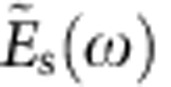
 and 
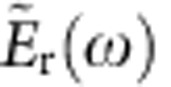
 are Fourier-transformed time-domain electric field transmitted through the sample and reference, respectively^27^,^28^. The open circles in Fig. 3a–c show the measured transmission spectra 
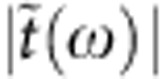
 through the GSTD sample at 0, −0.5, −1 and −3 V bias voltages under 140, 280 and 420 mW photoexcitation power, respectively. As graphene can be treated as a thin film, the transmission 
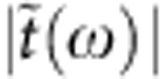
 can be simply expressed as^8^,^29^


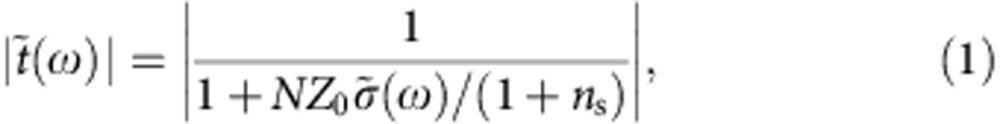


where, *N*=2 is the number of graphene layers, *Z*_0_ is the vacuum impedance, *n*_s_ is the effective refractive index of the substrate (see Methods) and (*ω*) is the complex sheet conductivity of graphene, which can be approximately described by the Drude model^9^,^30^


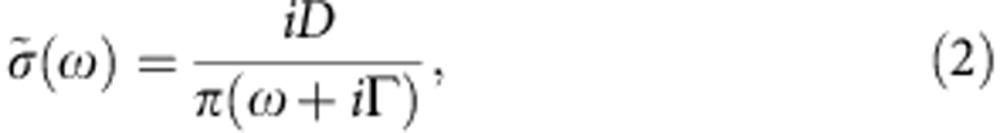


with *Γ* being the carrier-scattering rate and *D* being the Drude weight, and *D* can be further expressed as


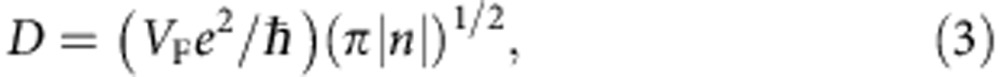


where, *V*_F_ (=1.1 × 10^6^ m s^−1^) is the Fermi velocity, *e* is the electron charge, *ℏ* is the reduced Planck constant and *n* is the carrier concentration.

**Figure 4:**
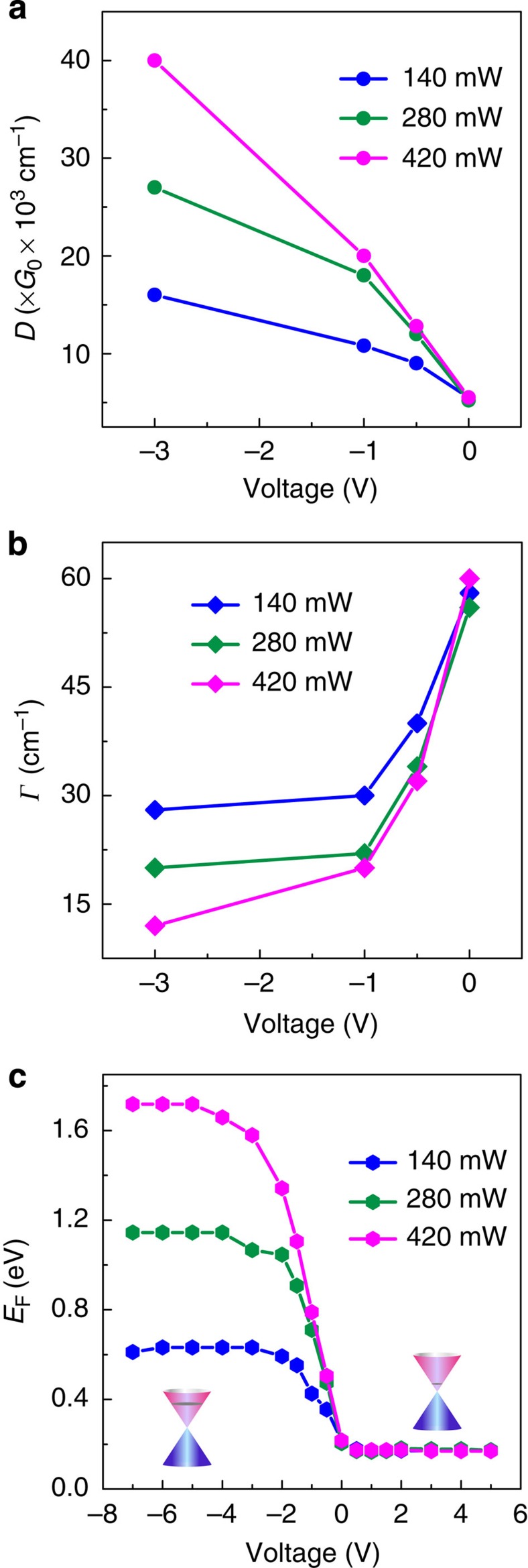
Theoretical values of *Γ*, *D* and *E*_F_. Calculated (**a**) Drude weight, and (**b**) carrier-scattering rate and (**c**) Fermi energy as a function of bias voltage at different photoexcitation power of 140, 280 and 420 mW, respectively. Insets in **c**: schematic diagram of the Fermi levels. On photoexcitation, the Fermi level is near the Dirac point in the conduction band at the positive bias, while the Fermi level moves away from the Dirac point to the higher conduction band at negative bias.

By fitting the amplitude transmission based on equations (1) and (2), we obtained the two important parameters of the graphene conductivity *D* and *Γ*. The solid black curves in Fig. 3a–c present the fitted amplitude transmission of the GSTD sample. A good agreement was achieved between the experiment and the theory. The fitted values of *D* and *Γ* are shown in Fig. 4a,b, respectively. It can be seen that the Drude weight *D* increased, whereas the scattering rate *Γ* decreased with an increase in the negative bias voltage. According to equation (3), the increase in Drude weight implies an increase in the carrier density. For example, at 280 mW photoexcitation and bias voltage of −1 V (henceforth ‘280 mW, −1 V'), the Drude weight and carrier-scattering rate are estimated to be *D*=18 × 10^3^ × *G*_0_ cm^−1^ and *Г*=22 cm^−1^, respectively, where *G*_0_=2*e*^2^/*h* is the quantum of conductance. By applying equation (3), the carrier density is estimated as *n*_1_=3.07 × 10^13^ cm^−2^. In contrast, the carrier density at 280 mW, −3 V is estimated out to be *n*_2_=6.90 × 10^13^ cm^−2^, which is more than twice of *n*_1_. The enhanced carrier density implied that a slight increase in the negative bias voltage (0 to −3 V) would inject a significant number of carriers into the graphene layer. It is also found that a clear enhancement in Drude weight appears with the reinforcement of the photoexcitation power under the same negative voltage due to an increase in the number of carriers excited in the silicon substrate. Whereas for the carrier-scattering rate, it indicates an inverse variation trend compared with the Drude weight, which is very interesting, as the larger carrier density normally reveals a higher scattering rate due to stronger carrier–carrier interactions. This behaviour could have been a result of the trap-filling effect due to impurities in the graphene layer, which inhibits the carrier recombination^31^. It is also worth noting that there is only a minor change in both the values of *D* and *Γ* when there is no applied bias voltage. This implies that the carrier diffusion from the photoexcited silicon only played a minor role in the carrier accumulation in graphene. The level of the fitted Drude weights and carrier-scattering rates also agrees well with the recently reported results^9^,^30^,^32^. Figure 4c illustrates the corresponding variation of the Fermi energy *E*_F_ in graphene extracted from 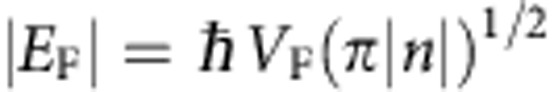, which clearly shows the variation in the Fermi energy under different photoexcitation power and biases.

## Discussion

To further elucidate the mechanism, we could interpret the behaviour of the GSTD sample as that of an electronic ‘PN' junction. As the silicon substrate used here is an *N*-type semiconductor, the graphene would act as a ‘*P*-type material' in this configuration. When the GSTD sample was photoexcited by the green laser, a photo-conductive silicon layer was generated beneath the graphene layer where the electron density was enhanced due to carrier generation. However, the amount of photoexcited carriers generated in the graphene layer was much smaller due to small optical absorption^17^. Thus, a density difference was formed at the graphene–silicon interface. As a result, the electrons would first diffuse from the photo-conductive silicon layer into the graphene film until an equilibrium was reached, resulting in the formation of a depletion layer with lower conductivity in the photo-conductive silicon layer (the graphene layer is quite thin). With the injection of the diffusion electrons, the holes in the graphene (chemical vapour deposition graphene is weakly hole doped) recombined with electrons and then an increased number of empty electron states were filled, hence, the initial Fermi level of the graphene under photoexcitation was in the conduction band at zero bias. Meanwhile, the graphene conductivity was increased, leading to attenuation of the terahertz transmission. At positive bias, the depletion layer became thinner. Beyond a threshold bias (∼0.5 V), the electrons could form a current flow in the electronic circuit loop and thus became hard to accumulate in graphene. Therefore, a small enhancement in transmission was observed when the voltage was increased from 0 to 0.5 V, and there was no obvious change in the transmission when the bias voltage was further increased from 0.5 to 5 V. On the other hand, when the negative bias voltage was applied, the diffusion of carriers was weakened, while the depletion layer was broadened. The ‘PN' junction behaved as a parallel plate capacitance. More electrons would be injected into the graphene layer as the negative bias increased, which shifted the Fermi level further away into higher conduction band. Meanwhile, the conductivity of graphene further increased, which led to higher attenuation of the terahertz wave. Further increase in the negative bias gave rise to saturation in the carrier density of the graphene layer. Thus, the terahertz transmission remained constant on any further increase in the negative bias up to −7 V. We found a saturation point in the terahertz transmission peak at the bias voltage of −3 V for all the three different photoexcitation power. At the saturated voltage bias, the transmission could only be tuned by varying the photoexcitation pump power where a larger power would result in a smaller transmission.

In the above analysis, we attribute the transmission modulation to the conductivity change in graphene; however, the conductivity of the photo-conductive silicon layer was also modified under the external bias, which could also contribute to the modulation of terahertz transmission at the same time. To validate the calculated results, transmission spectra of the GSTD were simulated using CST Microwave Studio, where the photo-conductive silicon layer was also taken into account (see Methods). It could be seen from the simulation results shown in Fig. 3d–f that the bias and optical excitation-dependent transmission spectra are in good agreement with the experimental data, which confirmed that the modulation of the amplitude transmission is essentially associated with change in the conductivity of the graphene thin film.

In summary, we have experimentally demonstrated a graphene–silicon hybrid film that behaves as an efficient ‘diode' for the terahertz waves under simultaneous CW photoexcitation and DC bias voltage. Compared with the existing electronically controlled devices, the GSTD achieves a large modulation depth of up to 83% at a small negative gate bias voltage of −4 V. Our work also highlights that graphene is compatible with silicon-based electronics and photonics. The active tuning behaviour of the graphene–silicon hybrid film would enable promising applications in terahertz technology, such as communications, plasmonic engineering and graphene-based metamaterials.

## Methods

### Theoretical fitting

The thickness (∼1 μm) of the photo-conductive silicon layer, which was generated below the graphene under photoexcitation, was determined by the penetration depth of the 532-nm laser in silicon^33^. The photo-conductive layer along with the unexcited silicon were treated as an effective substrate with a refractive index *n*_s_=3.45 when we fit the Drude weight *D* and scattering rate *Г* using equations (1) and (2). This was based on the fact that the thickness of this photo-conductive silicon layer is quite small compared with the terahertz wavelength, and meanwhile its conductivity/permittivity is also not sufficiently high to affect the effective refractive index.

### Numerical simulations

**Figure 5:**
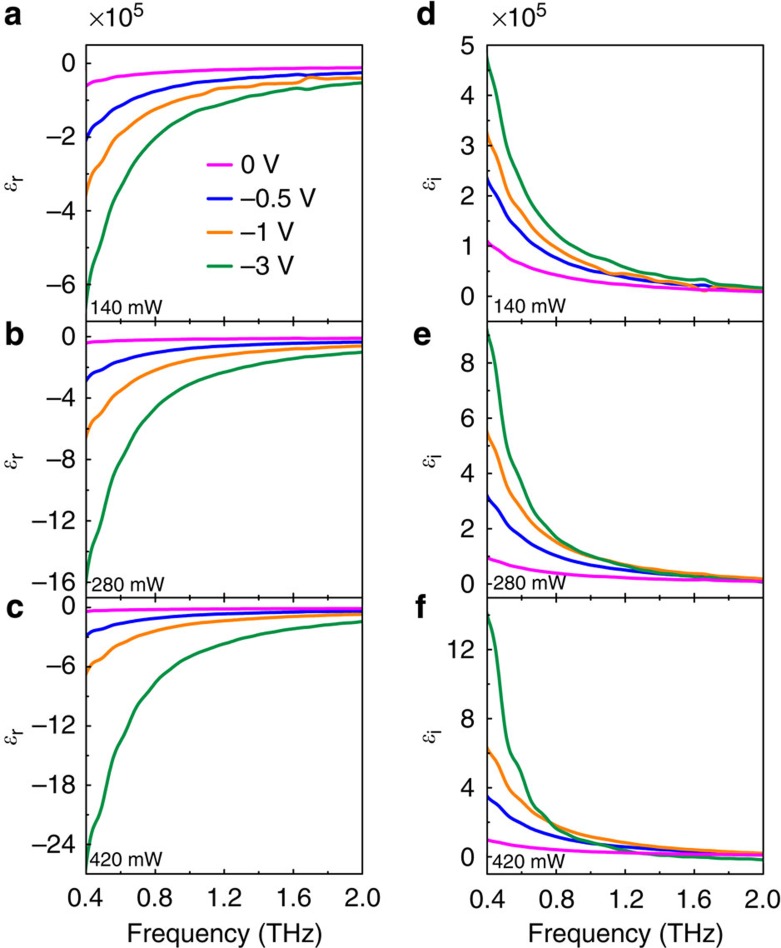
Theoretical values of graphene permittivity. (**a**–**c**) Calculated real parts of graphene permittivity as a function of frequency biased at 0, −0.5, −1 and −3 V with the photoexcitation power of 140, 280 and 420 mW, respectively. (**d**–**f**) Calculated imaginary parts of the graphene permittivity corresponding to **a**–**c**, respectively.

Finite-element frequency-domain method was carried out to simulate the transmission spectra using CST Microwave Studio. In the simulation, conductivity variation of the photo-conductive silicon layer under various photoexcitation power was taken into consideration. The conductivity of the photo-conductive silicon layer under 140, 280 and 420 mW photoexcitation was set to be 600, 1,200 and 1,800 S m^−1^, respectively, based on experimental measurements (Supplementary Fig. 4). When biased with negative voltages, the conductivity of the photo-conductive silicon layer decreased due to broadening of the depletion layer^34^. To find the maximum change in transmission the photo-conductive silicon layer has caused, the conductivity of the photo-conductive silicon layer was set to be unchanged at negative bias. Meanwhile, the double-layer graphene was modelled as a 2-nm-thick film and its permittivity =*ɛ*_r_+*iɛ*_i_ (as shown in Fig. 5) was calculated using the relations *ɛ*_r_=−*σ*_i_/(*ωdɛ*_0_) and *ɛ*_i_=*σ*_r_/(*ωdɛ*_0_)^14^,^35^, where its conductivity =*σ*_r_+*iσ*_i_ was extracted from equation (1), and d is the thickness of graphene.

## Author contributions

Q.L., Z.T., J.H. and W.Z. proposed the active graphene–silicon hybrid metamaterial. Q.L. and X.Z. completed numerical simulations. Q. L. and J.G. fabricated the graphene–silicon hybrid diode sample. Q.L. and Z.T. performed all the measurements. Q.L., X.Z., L.D. and Z.T. discussed the comparisons between simulations and experiments. Q.L., X.Z., L.D., Z.T., J.G. and R.S. analysed the measured data. J.H., R.S. and W.Z. supervised the theory and the measurements. All the authors discussed the results and contributed to the writing of the manuscript.

## Additional information

**How to cite this article:** Li, Q. *et al*. A graphene–silicon hybrid diode for terahertz waves. *Nat. Commun*. 6:7082 doi: 10.1038/ncomms8082 (2015).

## Supplementary Material

Supplementary Information Supplementary Figures 1-4 and Supplementary References
