# Supplementary material for: Active graphene–silicon hybrid diode for terahertz waves
Source: Nat Commun. 2015 May 11;6:7082. doi: 10.1038/ncomms8082 (PMC4432643; doi:10.1038/ncomms8082)
Supplement: Supplementary Information — Supplementary Figures 1-4 and Supplementary References [file ncomms8082-s1.pdf]

Supplementary Figures

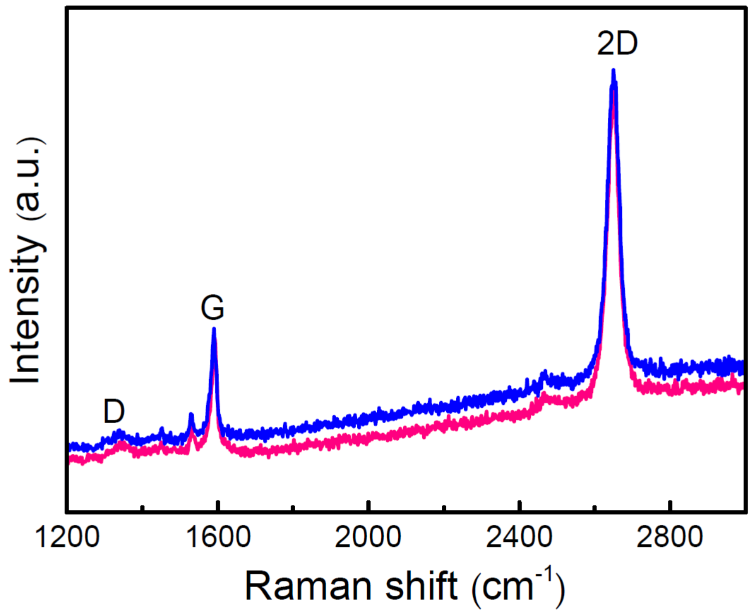

Supplementary Figure 1. Raman spectra of the double-layer graphene. Raman spectroscopy was performed on the double-layer graphene with a 633 nm excitation laser at two different locations. The D, G and 2D peaks were located at ~1340, 1589, and 2652 cm^{-1}, respectively. The ratio of the 2D peak to the G peak intensity (I_{2D}/I_G) is higher than 2, and the full width at half-maximum (FWHM) of the 2D peak is approximately 37 cm^{-1}. The Raman spectra show similar features to that of the monolayer graphene, which could be due to the small rotation angle between the two non-interacting graphene layers[1,2].

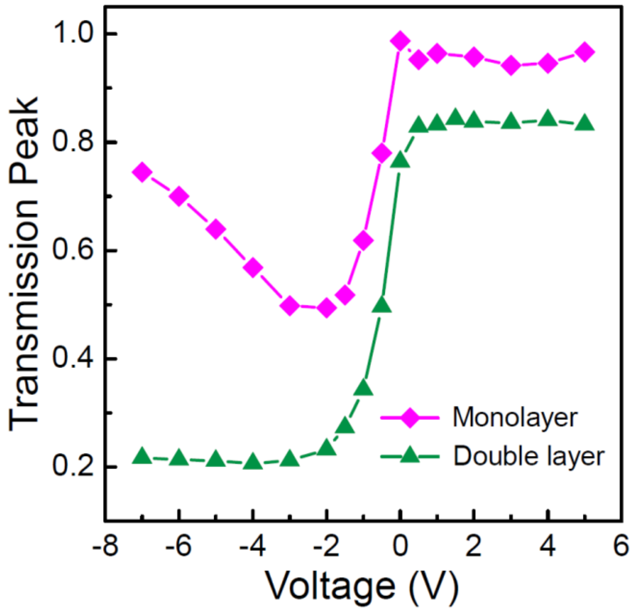

Supplementary Figure 2. Normalized transmission peaks of the GSTD devices with the monolayer and double-layer graphene. Normalized terahertz time-domain transmission peaks of the GSTD devices with the monolayer and double-layer graphene were measured at different bias voltages under 280 mW continuous wave laser excitation. In order to observe the difference between the monolayer and double-layer graphene, we also made a GSTD sample where the monolayer graphene was transferred onto an identical silicon substrate and measured the transmission properties under 280 mW photoexcitation and various electrical bias. It is seen that the modulation shows a quite similar trend when compared to that of the double-layer sample as described in the main text. The terahertz transmission changes slightly at positive bias voltage while dramatically decreases at negative bias. However, compared to the double-layer graphene, the modulation depth of the monolayer sample is ~50% at -3 V, which is much lower than what we achieved with the double-layer sample (~72% at 280 mW). The GSTD sample with the double-layer graphene illustrates a better “diode” performance under larger negative biases, while it is interesting to see that the transmission with the monolayer graphene starts to increase as the negative bias increases beyond -3 V after a small range of saturation (-2 to -3 V). This is caused due to the breakdown effect of the “PN” junction, whereby the electrons again formed an electric circle similar to that under positive biases. In this case, the electron could not be further accumulated in the graphene layer, thus the electron density of the graphene decreased, leading to increased terahertz transmission beyond -3 V. As the double-layer graphene could allow accommodation of higher electron density, the breakdown voltage is estimated to be higher than that of the single-layer graphene, which is out of the applied voltage range. Therefore, the breakdown effect was not observed in the double-layer GSTD device. Thus, the GSTD device with the double-layer graphene instead of the monolayer film was studied in the main text due to enhanced terahertz modulation and the improved diode performance.

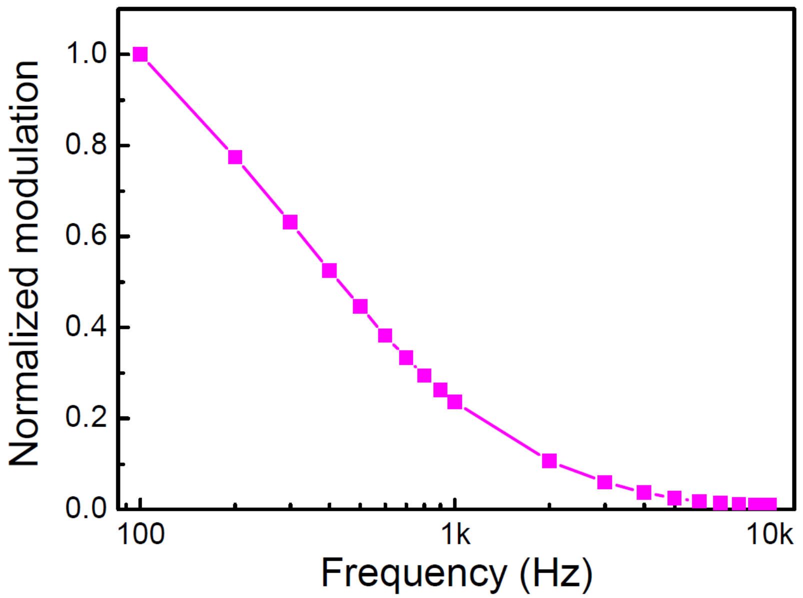

Supplementary Figure 3. Measured electrical modulation speed of the GSTD device. Measured electrical modulation speed of the GSTD device under -3 V – 0 V AC rectangular voltage bias and 280 mW continuous wave photoexcitation. To measure the modulation speed of the proposed GSTD device (double-layer graphene) under external bias, we applied an AC rectangular voltage (-3 V – 0 V) to the GSTD sample and swept the voltage frequency from 100 Hz to 10 kHz; this was a method used in previous studies[3,4]. By observing the modification in the peak value of the terahertz time-domain pulses, we estimate that the modulation speed of the GSTD device is approximately 1 kHz. The modulation speed could be further improved by reducing the RC time constant in the GSTD device, e.g. using substrate with higher doping level to decrease the resistance[4]. However, it should be noted that the substrate cannot be heavily doped since it would attenuate the terahertz transmission.

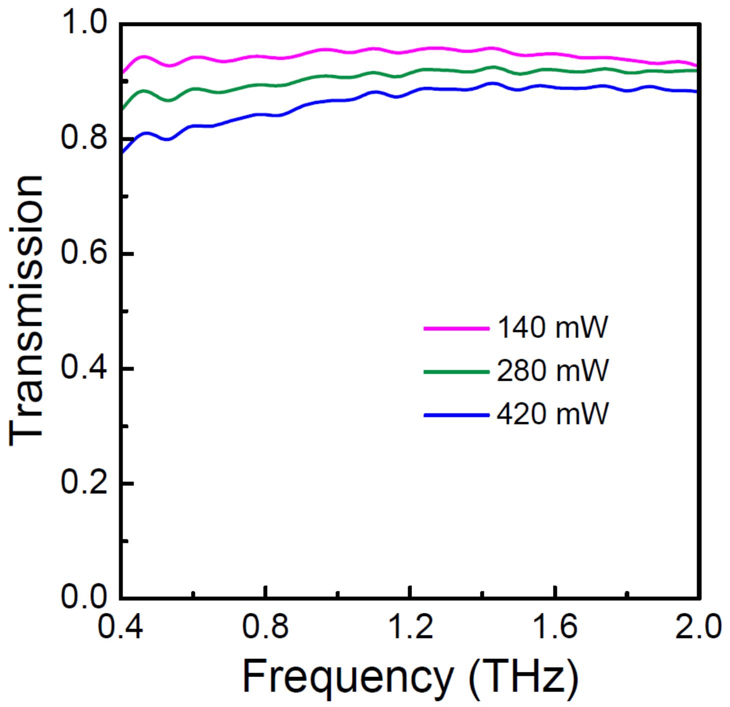

Supplementary Figure 4. Measured frequency-domain transmission of a bare silicon at various photoexcitation power. Measured terahertz transmission spectra of a bare silicon with continuous wave laser photoexcitation of 140, 280, and 420 mW, respectively. When the bare silicon is photoexcited by a continuous wave 532 nm laser, a photo-conductive silicon layer is formed. The thickness of the photo-conductive silicon layer is determined by the penetration depth of the incident laser, which is 1 µm in our case. The conductivity of the photo-conductive silicon layer is much higher than that of the bare silicon due to increased carrier density. The terahertz transmission decreases under photoexcitation. Here, we took the transmission of the identical bare silicon without photoexcitation as a reference. It can be seen that the transmission decreases with higher photoexcitation power due to enhanced carrier density in the photo-conductive silicon layer. From the measured result, the estimated conductivities of the 1-µm-thick photo-conductive silicon layer are 600, 1200, and 1800 Sm^{-1}, respectively, with photoexcitation power of 140, 280, and 420 mW.

Supplementary References

1. Ferrari, A. C., Meyer, J. C., Scardaci, V., Casiraghi, C., Lazzeri, M., Mauri, F., Piscanec, S., Jiang, D., Novoselov, K. S., Roth, S. & Geim, A. K., Raman spectrum of graphene and graphene layers. *Phys. Rev. Lett.* **97,** 187401 (2006).
2. Kim, K., Coh, S., Tan, L. Z., Regan, W., Yuk, J. M., Chatterjee, E., Crommie, M. F., Cohen, M. L., Louie, S. G. & Zettl, A. Raman spectroscopy study of rotated double-layer graphene: misorientation-angle dependence of electronic structure. *Phys. Rev. Lett.* **108,** 246103 (2012).
3. Lee, S. H., Choi, M., Kim, T.-T., Lee, S., Liu, M., Yin, X., Choi, H. K., Lee, S. S., Choi, C.-G., Choi, S.-Y., Zhang, X. & Min, B. Switching terahertz waves with gate-controlled active graphene metamaterials. *Nat. Mater.* **11,** 936-941 (2012).
4. Chen, H.-T., Padilla, W. J., Zide, J. M. O., Gossard, A. C., Taylor, A. J. & Averitt, R. D. Active terahertz metamaterial devices. *Nature* **444,** 597-600 (2006).
